# Survival of silver diamine fluoride among patients treated in community dental clinics: a naturalistic study

**DOI:** 10.1186/s12903-020-01379-x

**Published:** 2021-01-20

**Authors:** Sarah E. Raskin, Eric P. Tranby, Sharity Ludwig, Ilya Okunev, Julie Frantsve-Hawley, Sean Boynes

**Affiliations:** 1 L. Douglas Wilder School of Government and Public Affairs, Virginia Commonwealth University, Richmond, VA USA; 2 DentaQuest Partnership for Oral Health Advancement, Westborough, MA USA; 3 Advantage Dental, Redmond, OR USA

**Keywords:** Silver diamine fluoride, Dental caries, Aerosol, Community dentistry, Survival analysis, Kaplan–meier estimate, COVID-19

## Abstract

**Background:**

Silver diamine fluoride (SDF) is a minimally-invasive preventive service used in the U.S. to avert and arrest caries since 2014. No studies document survival outcomes based in real world delivery. We analyzed 12-month survival outcomes of SDF applied independently or concurrently with other restorative procedures among a population receiving community dental care.

**Methods:**

We analyzed data on SDF applications from de-identified dental claims on Oregon Health Plan patients served by Advantage Dental in 2016, who had been seen in 2015 (patient n = 2269; teeth n = 7787). We compared survival rates of SDF alone, SDF applied with a sedative filling, and SDF with a same-day restoration. Failure was defined as a restoration or extraction of the tooth 7 to 365 days after initial application. Survival was defined as a patient returning 180 or more days after application whose tooth did not have a restoration or extraction. Differences were assessed through Wilcoxon equality of survivor function tests and log-rank equality of survivor tests to compare failure rates, Cox Proportional Hazards models to assess factors associated with survival of SDF, and Kaplan–Meier survival estimate to calculate the probability of survival over time.

**Results:**

SDF alone had an overall survival rate of 76%. SDF placed with sedative filling and with a same-day restoration had survival rates of 50% and 84% respectively, likely reflecting treatment intent. SDF alone survived exceptionally well on primary cuspids, permanent molars, and permanent bicuspids and among patients aged 10 to 20 years, with modest variation across caries risk assessment categories. A single annual application of SDF was successful in 75% of cases. Among SDF failures on permanent dentition, more than two-thirds of teeth received a minor restoration.

**Conclusion:**

SDF is a minimally invasive non-aerosolizing option that prevented non-cavitated lesions and arrested early decay among community dentistry patients when applied independently or concurrently with restorative procedures. Professional organizations, policy makers, providers, and payors should broaden optional SDF use by informing clinical guidelines, reimbursement policies, and treatment decisions. Future research should address clinical, social, service delivery, workforce, and economic outcomes using diverse population-based samples, and the mechanisms underlying single application success and caries prevention potential.

## Background

Dental caries, the most common disease of childhood and most prevalent health condition worldwide, persists despite concerted clinical and public health efforts to eliminate it over the last half-century [1, 2]. Untreated caries contributes to health problems including pain, poor quality of life, and psychosocial suffering, as well as societal burdens that include reduced productivity at work and school [1, 3, 4]. Preventing dental caries through oral health education, home hygiene, the avoidance of fermentable carbohydrates, consumption of fluoridated water, and access to and utilization of routine dental screenings, examinations, and care is essential in reducing disease incidence and burden, meeting population-level oral health goals, and addressing patient concerns [1, 4]. Because dental caries is a progressive disease, treating it as early as possible can halt extant disease, prevent or forestall subsequent cases, improve the longevity of teeth and their supporting structures, and, when possible, help patients avoid more invasive procedures and associated risks [4, 5]. As in primary prevention, secondary approaches that leverage treatment-as-prevention are particularly valuable when implemented at the population level [1, 5, 6]. Dental public health and oral health stakeholders seek to maximize caries prevention and treatment approaches that are safe, simple, effective, low-cost, minimally invasive, and amenable to delivery in a variety of community settings and by multiple members of dental treatment teams.

Silver diamine fluoride (SDF) has been approved for dental use in numerous countries including, since 2014, the United States, where its off-label use for the secondary prevention of caries beginning in 2016 has been subsequently formalized through clinical guidelines released in 2018 [7–9]. SDF has gained prominence among other non-invasive treatments in arresting established caries, though effectiveness varies by frequency of application (e.g., annually vs. biannually), preparation of concentrations, tooth type (e.g. permanent versus primary), and tooth surface (e.g., coronal vs. root surfaces) [6, 10–17]. SDF has also been demonstrated to prevent new carious lesions on root surfaces among older adults, while limited evidence indicates its potential to prevent caries in primary teeth for at least 24 months following initial application [15, 18, 19]. Numerous characteristics of SDF reflect those valued in dental public health interventions, including being minimally invasive, affordable, portable, and appropriate for use at scale in community settings by various multiple dental and medical team members, outside of clinical applications. In addition, amidst the proliferation of the SARS-CoV-2 virus (COVID19), SDF has been recommended as an appropriate, non-surgical, non-aerosolizing caries management procedure that complies with guidance from public health officials, regulatory bodies, and professional associations to limit the risk of exposure to airborne pathogens [18–21]. Concerns regarding the staining effects of SDF potentially limit its desirability for use on anterior dentition [22, 23]. However, recent evidence also documents its acceptability among dentally underserved patient groups for use on posterior dentition, and when posited to parents as a safe, minimally invasive, and effective alternative to procedures that could be painful or for which their children might otherwise be sedated, with particular suitability for children with behavioral challenges, often surpassing provider preference for using SDF [24–29].

The existing literature on SDF focuses primarily on young children who still have primary dentition [10, 13, 15, 22–28] and older adults [16, 30], often omitting older children, adolescents, and working-age adults. The strongest evidence on SDF derives from randomized controlled trials whether individual or aggregated into evidence reviews, which compare SDF with placebo or other treatments, limit the intervention to SDF alone versus when used in combination with restorative procedures, and generate findings from samples treated under ideal clinical conditions and from analyses that control for covariates [6, 10–17]. While this evidence supports SDF effectiveness in arresting caries lesion development and progression, accounts or analyses of “real world” concerns such as the settings in which treatments are delivered, patient volume at scale, and clinical decision-making when multiple treatment options are available, are limited in literature, as is evidence of the potential for SDF to prevent caries.

This study aims to address some of these limitations, with particular concern for dentally underserved patients who obtain care in community settings and who are also historically excluded from clinical trials due to geographic and other barriers. It describes survival outcomes of SDF applied independently or concurrently with a sedative or restorative procedure among a population receiving care in community settings over the course of 1 year. Utilizing a retrospective analysis of patient claims filed with the largest dental accountable care organization in Oregon, this study explores variations in health service delivery to document SDF survival in a real-world community-based practice setting. To our knowledge, it is the first study to assess SDF survival among a population sample treated in a real-world practice setting, both when used alone and in combination with a sedative filling or restoration.

## Methods

We analyzed data on SDF applications from de-identified dental claims on Oregon Health Plan patients served by Advantage Dental in 2016, among patients who had been seen in 2015.

### Setting

Advantage Dental delivers services to approximately 284,000 members of the Oregon Health Plan, the state’s Medicaid program, and contracts with 14 of the 16 of the state’s coordinated care organizations. Operating in a value-based care design, Advantage prioritizes community care delivered by remote supervision dental hygienists, with an emphasis on disease prevention and management vis a vis outreach, assessment, preventive services, and referral to interprofessional oral healthcare. With regards to its patients, 3 out of 5 (61%) reside in rural areas of Oregon, predominately in the west and southern regions. The rest live in more urban areas, with 10% residing in Portland or near suburbs. All beneficiaries, and therefore all patients whose claims were analyzed for this study, meet state income guidelines for Medicaid enrollment: Adults with household incomes up to 138% of the federal poverty level, or $17,609 for an individual, children who reside in households with incomes up to 305% of the federal poverty level, or $52,582 for two members, and special populations such as pregnant women.

In 2016, Advantage incorporated twice-annual 38% SDF into its clinical guidelines as a risk-based treatment option, following an established protocol [7, 31–33]. The goal of increased adoption and utilization of SDF was to bolster efforts to reduce oral health disparities by optimizing community-based approaches with multi-disciplinary teams to arrest or prevent early stage caries disease [32]. Clinicians determined patients’ caries risk category by completing a four item chairside examination [33]. Patients exhibiting current cavitated lesions or signs of infection were categorized as being at high risk of caries. Among patients who did not have a cavitated lesion or sign of infection, assignment to the low or moderate caries risk group was determined by prior caries experience and visual changes in tooth structure (opacity or white, brown, or grey shadowing). Clinical guidelines indicated that patients determined to be at moderate risk of caries were eligible to receive twice-annual SDF to the occlusal surfaces of posterior teeth for preventive treatment of future lesions. Patients determined to be at high risk of caries were eligible to receive twice-annual preventive SDF as well as SDF application to stabilize cavitated lesions until definitive care could occur, with temporary restorations without excavation where appropriate. SDF was also made available to treat hypersensitivity. Chairside SDF application instructions followed a standardized six-step process that emphasized the importance of maintaining dryness on the lesion, restricting SDF only to the treatment area, preventing tissue staining, and providing patient instructions; interproximal lesions were reached with SDF using Super floss. SDF decision-making during treatment planning and placement on the tooth occurred at the discretion of the provider and the consent of the patient, thus creating the possibility of deviation from guidelines, a natural variation upon which this analysis is premised. Nearly 200 (199) providers applied SDF during the measurement period.

### Study population and data sources

The data used in this study comes from a retrospective review of dental claims data. The study population included all Advantage Dental patients age 0 to 64 who had at least one SDF application, defined as the presence of CDT code D1354 on a patient claim. Current Dental Terminology (CDT) codes are billing codes used to identify dental procedures in dental claims data. We analyzed teeth that were treated with an SDF application and sedative filling (D2940/D2941) on the same day or SDF application and restoration on the same day separately from teeth that received only an SDF application (See Additional file 1: Appendix 1). We assessed survival of SDF among teeth that had been tracked for at least 365 days. As a result, the survival analysis only included patients with teeth that had an initial application of SDF treatment in 2016 and for which the tooth number was identified. To reduce the potential for survival to be affected by right-censoring, patients were only included in the cohort if they had been a patient in 2015. Third molars were excluded from the analysis, as were teeth in which both SDF and a sealant were placed on the same day and teeth for which a remnant was removed. We limited the sample age range to 64 years old in order to limit the potential for our analysis to conflate use of SDF for caries prevention and arrest versus for hypersensitivity, which can become more common as patients age [34].

### Variables and measurement

SDF treatments were considered to have survived if the patient was seen by Advantage Dental at least once 180 days or later after the initial application and the treated tooth did not have a failure. Failures were defined as the treated tooth receiving any restoration, endodontic treatment, or extraction seven or more days after initial application, except for sedative filling/protective restorations (D2940/D2941) if they occurred within 10 weeks of the initial application. Teeth were tracked for failure for at least 365 days, and up to 720 days, after application.

We assessed variations in application and survival by demographics, in particular, age group. We examined survival by primary versus permanent teeth and location in the mouth (lower and upper incisors, cuspids, bicuspids, and molars). Clinical guidelines indicate SDF use for patients at high risk for caries. Therefore, we also compared variation by caries risk. Caries risk was assessed chairside using visual techniques guided by three clinical screening questions and one patient history question, and recorded within claims records as D0601 (low caries risk), D0602 (moderate caries risk), and D0603 (high caries risk). If multiple assessment scores were reported, we used the first reported risk assessment in the period of the study.

### Statistical methods

We conducted a descriptive analysis and used various statistical methods in the analysis. All analyses were done in Stata 16. We used Wilcoxon equality of survivor function tests and log-rank equality of survivor tests to determine differences in overall survival rates between types of SDF applications. We used Cox Proportional Hazards models to assess the factors associated with survival of SDF. These models use robust clustered standard errors to correct for the non-independence of standard errors to account for the multilevel design of the data, in which teeth are nested in patients. Estimates are reported in the hazard ratio (HR) and their respective 95% confidence interval (95% CI). The Efron method was used to handle tied failures. Finally, we used Kaplan–Meier methods to calculate the probability of survival over time.

### Reporting

We prepared this manuscript by following STROBE guidelines.

## Results

### Description of study participants

Overall, 7787 teeth from 2269 patients were included in the study. We excluded from the sample patients from the overall beneficiary group whose records were missing data (See Additional file 1: Appendix 2). The majority of patients (91%) received only SDF, with 7475 teeth receiving SDF alone (2063 patients), 220 receiving both an SDF application and a sedative filling (185 patients), and 92 receiving SDF with a same-day restoration (76 patient, Table 1). Study participants were well-distributed by age, ranging from 1 to 64 years. The study sample disproportionately included the youngest beneficiaries relative to the overall age distribution, likely due to the high prevalence of delivery of these treatments to children (See Additional file 1: Appendix 2).

**Table 1:** Descriptive statistics of teeth treated with silver diamine fluoride only or combined with sedative filling or restoration

	SDF		SDF + sedative filling		SDF + restoration	
	Count	%	Count	%	Count	%
Total	7475		220		92	
Age						
1–5	1696	23	45	20	5	5
6–9	2374	32	81	37	23	25
10–14	923	12	43	20	12	13
15–20	641	9	7	3	6	7
21–30	578	8	13	6	13	14
31–40	427	6	13	6	17	18
41–50	303	4	9	4	4	4
51–64	533	7	9	4	12	13
Caries risk						
No assessment	1723	23	75	34	18	20
Low	401	5	12	5	4	4
Moderate	1331	18	35	16	17	18
High	4020	54	98	45	53	58
# of Applications within 1 year						
1	4567	61	186	85	82	89
2	1827	24	25	11	8	9
3+	1081	14	9	4	2	2
Primary versus permanent						
Primary	4152	56	131	60	27	29
Permanent	3323	44	89	40	65	71
Tooth type—primary teeth						
Lower incisor	35	1	0	0	0	0
Lower cuspid	59	1	0	0	0	0
Lower molar	1801	43	72	55	15	56
Upper incisor	350	8	1	1	2	7
Upper cuspid	161	4	1	1	1	4
Upper molar	1746	42	57	44	9	33
Tooth type—permanent teeth						
Lower incisor	84	3	0	0	0	0
Lower cuspid	86	3	1	1	1	2
Lower bicuspid	490	15	7	8	4	6
Lower molar	1001	30	31	35	21	32
Upper incisor	160	5	1	1	1	2
Upper cuspid	85	3	3	3	1	2
Upper bicuspid	514	15	8	9	12	18
Upper molar	903	27	38	43	25	38

Participants aged 1 to 20 accounted for the majority of all SDF delivered (75%, Table 1). The provision of SDF generally tapered as adults progressed through working age. Half of SDF with a same-day restoration were among adults 21 and older. As expected, SDF was commonly applied to those at increased caries risk, with 45–58% of applications delivered to patients assessed to be at high caries risk and 23–34% delivered to patients who did not have a caries risk assessment within the claims record. By contrast, only 4–5% of SDF was delivered to low-risk patients.

SDF alone and SDF with a sedative filling were more often placed on primary teeth than on permanent teeth, primarily on molars (43% and 55% on lower molars, respectively, and 42% and 44% on upper, Table 1). The remaining SDF-alone placements on primary teeth were applied primarily to upper incisors and upper cuspids (8% and 4%, respectively). SDF with a same-day restoration was more often placed on permanent teeth than on primary teeth and, in particular, permanent molars (32%-38%) and upper bicuspids (18%). When SDF was applied alone at the index visit, just over a third of teeth received one or more additional SDF applications within 1 year (38%). An analysis of the relationship between caries risk and number of SDF applications not included in this paper shows that single applications of SDF were more common among patients whose claims record did not contain a risk assessment than among patients whose claims record contained a risk assessment (64% versus 58%, respectively). Multiple applications of SDF during the study period (three or more) were marginally more common among patients assessed to be at high risk of caries than among patients assessed to be at low or moderate risk (17% versus 13%, respectively).

### Survival analyses

**Fig. 1:**
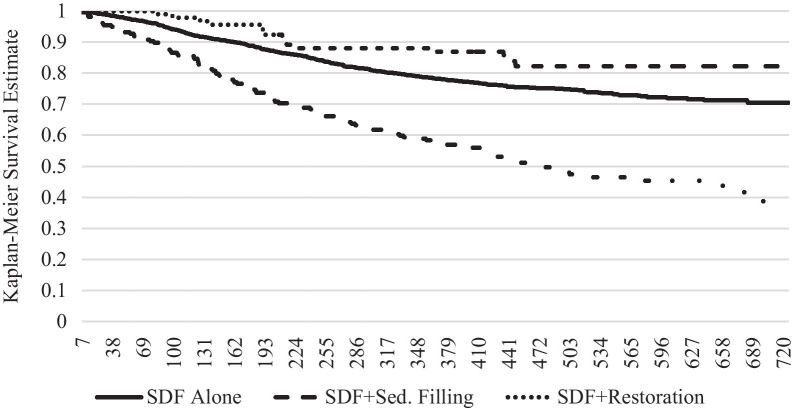
Kaplan–Meier survival plot

SDF alone had an overall survival rate of 76%, while SDF with a sedative filling had a survival rate of 50% and SDF with a same-day restoration has a survival rate of 84%. These survival rates are significantly different with both the Wilcoxon and Log-Rank tests (Table 2). Kaplan–Meier estimates of SDF survival alone and with a restoration remained above 90% survival to 162 days and 215 days, respectively, with SDF alone holding its overall survival rate of 76% well beyond a year, to day 446 (Fig. 1). SDF applied with a sedative filling fell below 90% survival at day 80 and remained at 58% at 1 year after application.

**Table 2:** Comparisons of survival rates of teeth treated with SDF applications alone versus SDF application with same day restoration among advantage dental patients 64 and under

	SDF Survival %	SDF + Sedative Filling Survival %	SDF + Restoration Survival %
Overall	76%	50%	84%
# of SDF applications within 1 year			
1	75%	49%	84%
2	77%	56%	–
3+	75%	33%	–
Caries risk			
Low	81%	50%	–
Moderate	76%	51%	82%
High	75%	42%	79%
No assessment	75%	59%	94%
Age			
1–5	69%	53%	–
6–9	77%	57%	61%
10–14	84%	53%	83%
15–20	82%	29%	100%
21–30	76%	23%	92%
31–40	77%	31%	100%
41–50	72%	33%	50%
51–64	68%	44%	92%
Primary versus permanent			
Primary tooth	74%	56%	74%
Permanent tooth	78%	40%	88%
Tooth type—primary teeth			
Lower incisor	74%	–	–
Lower cuspid	86%	–	–
Lower molar	71%	51%	73%
Upper incisor	77%	–	–
Upper cuspid	83%	–	–
Upper molar	75%	60%	67%
Tooth type—permanent teeth			
Lower incisor	70%	–	–
Lower cuspid	69%	–	–
Lower bicuspid	82%	29%	100%
Lower molar	80%	39%	81%
Upper incisor	50%	–	–
Upper cuspid	69%	–	–
Upper bicuspid	75%	–	100%
Upper molar	82%	47%	88%

Cells are empy if sample size is less than 10. Failure is defined as a restoration or extraction after application. Sedative fillings are not considered to be failures if they occur within 70 days of initial application. Survival is defined as a patient that returned 180 or more days after application and had no restoration or extraction. Equality of Survivor Tests for Overall Rate Between SDF Applications: Wilcoxon Test, 79 (2 df), *p* < 0.000; Log-Rank Test, 91.5 (2 df), *p* < 0.000)

SDF survival varied little based on the number of applications. However, there is substantial variation across other categories (Table 2). SDF alone survived well on all primary teeth with lower molars (71%) having the lowest survival rate and lower and upper primary cuspids (86% and 83%, respectively) performing well. SDF alone performed well on permanent molars (80–82%) and bicuspids (82–75%), with lower survival rates on incisors and cuspids. SDF survival rates also varied across age categories. SDF survival among patients aged 6 to 40 met or exceed the overall rate (76–84%), with survival particularly strong among those aged 10–20 years (82-84%). Survival rates were substantially lower among young children and adults age 41 and older. With regard to risk assessment, SDF survival was highest among patients with a low risk assessment (81%), while patients with a moderate risk assessment met the overall survival rate (76%). SDF survival among patients among patients with a high-risk assessment or lacking a risk assessment in their record fell short of the overall survival rate by one percentage point (75%). While this analysis presents results by tooth, outcomes at the patient level are consistent with tooth level, with survival rates of 72%, 47%, and 85% of SDF alone, SDF with a sedative filling, and SDF with a same-day restoration, respectively.

Among those with SDF applied with a sedative filling, survival rates by category never exceeded 60%. Moreover, SDF with a sedative filling failed at 2.5 times the rate of SDF alone, even after controlling for number of applications, caries risk, age, and tooth type and location and accounting for the multilevel design of the data (HR 2.49, *p* < 0.001, Table 3). This finding most likely is reflective of the interim nature of sedative fillings in current dental practice. Survival rates were highest among those under age 14 and lowest among patients between 15 and 50 years of age. SDF applied with a same-day restoration had the overall highest survival rates, with some categories reaching 100%, although the small sample size (n = 92) is important to keep in mind as is the intended longevity of the procedure and the potential selection bias of application only to teeth likely to succeed with this treatment.

**Table 3:** Cox proportional hazards regressions estimating silver diamine fluoride failure among advantage dental patients 64 and under

	Haz. Ratio	95% C.I	Robust clustered S.E
Type of application (reference: SDF alone)			
SDF + sedative filling survival %	2.48***	2–3.06	0.27
SDF + restoration survival %	0.60	0.33–1.08	0.18
# of SDF applications (reference: one)			
2	0.93	0.78–1.11	0.08
3	0.98	0.77–1.24	0.12
Caries risk (reference: low)			
No assessment	1.36	0.95–1.93	0.24
Moderate	1.34	0.92–1.94	0.25
High	1.42***	1.03–1.97	0.24
Age (reference: 6–9)			
1–5	1.33**	1.07–1.65	0.15
10–14	0.77	0.57–1.02	0.11
15–20	0.94	0.62–1.43	0.20
21–30	1.37	0.93–2.03	0.27
31–40	1.29	0.82–2.05	0.30
41–50	1.66*	1.09–2.55	0.36
51–64	1.79***	1.23–2.62	0.35
Tooth type—primary teeth (reference: lower molar)			
Lower incisor	0.66	0.23–1.91	0.36
Lower cuspid	0.40*	0.18–0.89	0.16
Upper incisor	0.61**	0.43–0.87	0.11
Upper cuspid	0.51**	0.33–0.78	0.11
Upper molar	0.86*	0.75–0.98	0.06
Tooth type—permanent teeth (reference: lower molar)			
Lower incisor	1.22	0.8–1.86	0.26
Lower cuspid	1.17	0.72–1.88	0.28
Lower bicuspid	0.77*	0.6–0.98	0.10
Upper incisor	2.65***	1.89–3.72	0.46
Upper cuspid	1.35	0.89–2.04	0.28
Upper bicuspid	1.17	0.9–1.52	0.16
Upper molar	0.86	0.7–1.06	0.09
Number of teeth	7787		
Number of patients	1957		
Likelihood ratio chi-square	154.48***	22 *df*	

*p < .05; **p < .01; ***p < .001

SDF applied to patients who had a high caries risk was likely to fail at approximately one-and-a-half times the rate of SDF applied to low-risk patients (HR 1.42, *p* < 0.001). SDF was significantly more likely to fail among patients aged 1–5 years and older than 41 years of age, when compared with those aged 6–9. On primary teeth, lower molars had significant higher rates of failure than other teeth, but no statistically significant difference from lower incisors. On permanent teeth, lower bicuspids were about 25% less likely to fail than lower molars, while upper incisors were about 2.7 times more likely to fail (HR 0.77, *p* < 05 and HR 2.65, *p* < 0.001). There were no significant differences in failure rates by number of applications.

This study also assessed procedures performed on teeth on which SDF failed (Table 4). When SDF was applied alone, minor restorations were the most common procedures overall, performed on 39% of all failed applications, followed by major restorations (29%) and extractions (21%). Among primary teeth, the most common procedure following an SDF failure among teeth treated with SDF alone and in combination with a same-day sedative filling was a major restoration (43% and 66% respectively), while among permanent teeth it was a minor restoration (68% and 70%). When combined with a same-day restoration, the most common procedure associated with a failure was a minor restoration. The permanent teeth most commonly extracted following SDF alone were upper bicuspids (27%), although this accounts for a fairly small number of cases (n = 26). Among the permanent tooth type with the most instances of SDF failure alone (lower molars, n = 204), minor restorations remained the most common subsequent procedure (65%), followed by extractions (21%), and major restorations (12%). Endodontic procedures were relatively uncommon across the entire sample.

**Table 4:** Procedures performed on teeth that fail after SDF application among advantage dental patients 64 and under

	Panel A: SDF alone										
	Overall	Permanent versus primary		Tooth type—permanent teeth							
		Prim	Perm	L. Incisor	L. Cuspid	L. Bicuspid	L. Molar	U. Incisor	U. Cuspid	U. Bicuspid	U. Molar
Minor restoration	39%	36%	68%	64%	74%	68%	65%	68%	69%	70%	69%
Major restoration	29%	43%	8%	8%	0%	10%	12%	6%	4%	4%	6%
Endodontics	1%	0%	2%	4%	7%	1%	1%	1%	0%	3%	1%
Extraction	21%	20%	22%	16%	19%	20%	21%	25%	27%	21%	23%
Other	0%	0%	1%	8%	0%	1%	0%	0%	0%	2%	0%
Total	1831	1094	737	25	27	87	204	80	26	128	160

	Panel B: SDF + sedative filling					Panel C: SDF + restoration		
	Overall	Permanent versus primary		Tooth type—permanent teeth		Overall	Permanent versus primary	
		Prim	Perm	L. Molar	U. Molar		Prim	Perm
Minor restoration	39%	10%	70%	53%	80%	60%	71%	50%
Major restoration	41%	66%	15%	21%	10%	13%	29%	0%
Endodontics	3%	0%	6%	5%	5%	13%	0%	25%
Extraction	17%	24%	9%	21%	5%	13%	0%	25%
Other	0%	0%	0%	0%	0%	0%	0%	0%
Total	111	58	53	19	20	15	7	8

## Discussion

This study analyzed data from claims filed with a large dental accountable care organization to describe survival outcomes of SDF applied independently or concurrently with other restorative procedures among a population receiving care in community settings over the course of 1 year. To our knowledge, this is the first study to examine population-level SDF survival in a real-world context characterized by in situ treatment decision-making and is one of the few studies to examine 12-month SDF survival among older children, adolescents, working-age adults, and patients prioritized in community dental outreach other than school-based settings. Overall, our findings support previous conclusions that SDF is an effective treatment that arrests caries among numerous tooth types and patient demographic groups, both when used with a sedative or permanent restoration and when used alone [6, 10–17]. Our findings also reveal limitations in the translation of clinical research on SDF into community practice settings that contribute to knowledge gaps with rate of applications and service delivery [8, 9]. Study results should be considered in the context of how SDF can expand opportunities for preventive care toward addressing the social determinants of oral health and achieving oral health equity. SDF can be used by multiple medical and dental providers working in diverse and sometimes unideal community settings to slow progression of disease and extend the time needed to complete a treatment plan among patient populations who are unable to consistently utilize care due to transportation, out-of-pocket cost, or other resource limitations; geographic distance and migration impediments; and other barriers to care [10, 11].

Our study finds merit in using SDF alone to prevent non-cavitated lesions and arrest early decay on primary teeth (in particular upper primary incisors and upper and lower primary cuspids) and on permanent teeth (in particular, lower permanent bicuspids) including children’s permanent teeth, in contrast to other recent findings [16]. We also found, consistent with other studies [e.g. 11, 16], limitations in SDF survival when applied to lower primary molars, among patients assessed to be at high risk of caries, among young children and adults age 41 and older, and when SDF was used together with a sedative filling.

Importantly, we found that a single application of SDF in a year can arrest caries or non-cavitated lesions, a result that is consistent with one recent study [6], that addresses evidence limitations identified in other studies [11, 15], and that contrasts other literature and practice guidelines [8, 9, 12], including the practice guidelines informing the data analyzed in this study [33]. Equally important, the modest difference found in SDF survival across patient risk categories indicates the merit of using SDF across all risk categories, in particular as contrasted with other oral health treatments whose survival is more dramatically differentiated by risk assessment categories [38, 39]. The finding that when SDF applied alone failed, minor restorations were the most common procedures overall (39%) and in particular among permanent teeth (68%) also indicates the potential of SDF to contribute to overall oral health by helping patients avoid or delay more invasive procedures and support the longevity of existing teeth and their supporting structures. Similarly, the modest survival of SDF placed with a sedative filling suggests its potential interim utility to “calm” a tooth with more evident decay and manage it through behavioral modification in order to forestall or avoid a subsequent invasive procedure.

Our overall findings may help providers confidently integrate SDF among the complementary services available to fulfill the full spectrum of caries and non-cavitated lesion prevention in primary and permanent dentition. The results are strengthened by the study’s design as a “natural experiment,” which utilizes the variations commonly found in health service delivery to facilitate comparisons. SDF may also meaningfully remediate the limitations of practice guidelines and payor norms, for example the application of sealants only to pristine permanent molars, commonly among school-aged children [35, 36].

Our findings take on additional importance in light of the COVID19 pandemic and the immediate- and longer-term transformations in dental service delivery changes necessitated to minimize risk for transmission of airborne pathogens [18–21]. SDF is a key preventive and therapeutic caries management technique in the dental care armamentarium to minimize aerosols in the dental setting. The COVID19 pandemic has illuminated a critical gap in the dental infection control standards that are not adequately poised to implement transmission-based precautions to address threat of air-borne pathogens. Thus, there is an immediate—and likely long-standing—need to reduce aerosol-generating procedures in the management of oral disease to minimize patient-to-patient transmission of SARS-CoV-2, to protect dental health care workers from harm, and to address in the long term a movement toward minimizing aerosol-generating procedures in dentistry.

### Future research directions

This study identifies meaningful knowledge gaps that should be addressed through future research. Targeted clinical studies should address the use of SDF to prevent early carious lesions; drivers of risk-stratified SDF outcomes; predictors of survival of a single application of SDF, with particular emphasis on treating non-cavitated lesions; and the relative effectiveness of using SDF alone versus in combination with same day restorations or sedative fillings. Community-based studies should assess SDF longevity beyond 1 year; survival of SDF when used in combination with other treatments not documented in this study such as dental sealants [37, 38]; predictors of SDF survival and post-failure procedures using data that permits analyses by patient diagnosis, provider type and consistency; and other data not available for this study; relationships between community-based delivery of SDF and social determinants of health; and economic outcomes such as societal costs deferred by SDF treatment. We also encourage more implementation research to understand factors associated with the implementation and uptake of SDF in diverse community settings, in particular research that documents patient-centered outcomes such as patient acceptability of SDF as well as clinical outcomes [23–29, 39].

### Limitations

This study has several limitations. Outcomes were only assessed among patients with a follow-up visit at a minimum of 180 days after initial treatment to ensure that teeth did not appear to survive due to the patient failing to return to the dentist. Some meaningful outcomes could not be assessed due to dentistry’s convention of not including diagnostic codes in claims data including: Outcomes by individual diagnosis (e.g., non-cavitated carious lesions versus cavitated lesions, hypersensitivity versus nascent decay, failure determinations), proximity of the treated tooth to other teeth, rationale for providing only one SDF application in a year, or effects of having the same clinician perform the evaluation, treatment, and/or restoration at a single or multiple points in time. In particular, this limitation prohibited us from making causal claims regarding the use of SDF to prevent caries in primary dentition versus to arrest disease despite our results indicating this outcome and from assessing the impact of patient preference on SDF use on anterior dentition versus posterior dentition. [22–28]. Because our interest is in a treatments suitable for primary prevention or the secondary prevention-oriented treatment of dental lesions and extremely early stage caries, our study likely sampled a healthier population, and should not be considered generalizable to a population with more advanced dental caries. We were also unable to stratify outcomes by socioeconomic status (SES) given the relative SES homogeneity of Medicaid-eligible populations and the lack of specific SES measures in patients’ claims records. It should also be noted that the utilization of SDF and its coding for benefit practices were new to most of the provider group represented in this analysis. When recorded, most SDF applications in this study were applied to the occlusal surface of teeth, as is expected, however tooth surface was not consistently recorded in the claims data so more work may be necessary to ascertain its effectiveness by surfaces of teeth. This study also did not follow teeth for longer than 2 years, so additional research may be needed to address the long-term efficacy of SDF.

## Conclusion

This study finds that silver diamine fluoride applied independently or concurrently with a sedative or restorative procedure is an effective treatment that prevented non-cavitated lesions and arrested early decay among numerous tooth types and patient demographic groups among a population receiving care in community settings over the course of 1 year. SDF is a valuable option for preventing or arresting early stage dental caries that can improve patient and population-level oral health and that complies with immediate- and long-term dental service delivery transformations to maintain patient care while minimizing risk for transmission of airborne pathogens. Professional organizations, policy makers, dental and medical providers, dental payors, and patients themselves should consider the relative success of this treatment in informing clinical practice guidelines, reimbursement policies, and treatment decisions, while also exercising cautions due to the limitations of this study. Future research should address clinical, social, health service delivery, workforce, and economic outcomes including costs and invasive procedures deferred, using diverse population-based samples.

## Supplementary Information

**Additional file 1**. Survival of silver diamine fluoride as a non-aerosol generating procedure among patients treated in community dental clinics.

## Data Availability

The datasets used and/or analyzed during the current study are available from the corresponding author on reasonable request and subject to compliance with patient privacy regulations.

## Abbreviations

ADA: American Dental Association
SDF: Silver Diamine Fluoride
