# Supplementary material for: Survival of silver diamine fluoride among patients treated in community dental clinics: a naturalistic study
Source: BMC Oral Health. 2021 Jan 20;21:35. doi: 10.1186/s12903-020-01379-x (PMC7816144; doi:10.1186/s12903-020-01379-x)
Supplement: Supplementary file 1 — Additional file 1. Survival of silver diamine fluoride as a non-aerosol generating procedure among patients treated in community dental clinics. [file 12903_2020_1379_MOESM1_ESM.docx]

**Supplemental Files for “Survival of silver diamine fluoride as a non-aerosol generating procedure among patients treated in community dental clinics”**

| **Appendix 1: Restorations that Occur on the Same Day as Initial SDF Application** | | | | | |
| --- | --- | --- | --- | --- | --- |
| Code | Description | Count | % | # of Failures | Survival Rate |
| Sedative Filling | |  |  |  |  |
| D2940 | Protective restoration | 215 | 69% | 110 | 49% |
| D2941 | Interim therapeutic restoration – primary dentition | 5 | 2% | 1 | 80% |
| *Subtotal* |  | *220* |  | *111* | *50%* |
| Restorations | |  |  |  |  |
| D2140 | Amalgam - one surface, primary or permanent | 15 | 5% | 1 | 93% |
| D2150 | Amalgam - two surfaces, primary or permanent | 21 | 7% | 3 | 86% |
| D2160 | Amalgam - three surfaces, primary or permanent | 11 | 4% | 0 | 100% |
| D2161 | Amalgam - four or more surfaces, primary or permanent | 2 | 1% | 0 | 100% |
| D2330 | Resin-based composite - one surface, anterior | 2 | 1% | 0 | 100% |
| D2331 | Resin-based composite - two surfaces, anterior | 3 | 1% | 0 | 100% |
| D2335 | Resin-based composite - four or more surfaces or involving incisal angle (anterior) | 1 | 0% | 1 | 0% |
| D2391 | Resin-based composite - one surface, posterior | 18 | 6% | 6 | 67% |
| D2392 | Resin-based composite - two surfaces, posterior | 15 | 5% | 3 | 80% |
| D2393 | Resin-based composite - three surfaces, posterior | 4 | 1% | 1 | 75% |
| *Subtotal* |  | *92* |  | *15* | *84%* |
| Total |  | **312** | **--** | **126** | **60%** |

| **Appendix 2: Demographics of Silver Diamine Fluoride Patients 64 and Under in Advantage Dental 2016** | | | | | |
| --- | --- | --- | --- | --- | --- |
| Age | | SDF Alone | SDF + Sedative Filling | SDF + Rest. | All Advantage Dental Patients |
|  | 0-5 | 23% | 20% | 5% | 14% |
|  | 6-9 | 32% | 37% | 25% | 14% |
|  | 10-14 | 12% | 20% | 13% | 14% |
|  | 15-20 | 9% | 3% | 7% | 10% |
|  | 21-30 | 8% | 6% | 14% | 14% |
|  | 31-40 | 6% | 6% | 18% | 12% |
|  | 41-50 | 4% | 4% | 4% | 9% |
|  | 51-64 | 7% | 4% | 13% | 13% |
| Sex | |  |  |  |  |
|  | Male | 51% | 51% | 70% | 55% |
|  | Female | 49% | 49% | 30% | 45% |
| Race | |  |  |  |  |
|  | White | 40% | 33% | 38% | 41% |
|  | Black | 0% | 0% | 4% | 1% |
|  | Hispanic | 7% | 2% | 4% | 5% |
|  | Other | 0% | 0% | 2% | 1% |
|  | No Race Recorded | 52% | 65% | 51% | 52% |
| Note: Percentages are calculated within each treatment type and demographic group | | | | | |
